# Editorial: SUMO Proteins in Host Defense

**DOI:** 10.3389/fcell.2021.789877

**Published:** 2021-11-03

**Authors:** Carmen Rivas

**Affiliations:** ^1^Centro de Investigación en Medicina Molecular y Enfermedades Crónicas (CIMUS), Instituto de Investigaciones Sanitarias (IDIS), Universidade de Santiago de Compostela, Santiago de Compostela, Spain; ^2^Centro Nacional de Biotecnología (CNB), Consejo Superior de Investigaciones Cient (CSIC), Madrid, Spain

**Keywords:** SUMOylation, SIM domain, innate immunity, PML, ubiquitin-like proteins

Post-translational modification SUMOylation is an important modulator of protein function and plays a critical role in different biological processes including regulation of innate immune and inflammatory responses. SUMO modulates the activity of the NF-κB signaling pathway, a master regulator of innate immune responses, at different levels. Shi et al. studied the modulation of NF-κB as well IRF1 by the E3 ubiquitin and SUMO ligase breast cancer-associated gene 2 (BCA2) and they show that BCA2 inhibits NF-κB, and activates or inhibits IRF1 depending on the cellular context. The relevance of SUMOylation in the modulation of innate immune pathways is highlighted by the development by pathogens of strategies to interfere with the SUMOylation pathway in order to avoid an efficient host immune response. An overview of the crosstalk between SUMO and immune pathways, as well as the ability of viral pathogens to target the SUMOylation pathway is provided by Sajeev et al..

Many SUMO substrates can interact with SUMO in a non-covalent manner through SUMO-interacting motifs (SIMs). Non-covalent interaction between SUMO or SUMOylated proteins and SUMOylation substrates has important regulatory consequences as shown by Tripathi et al. who demonstrate that the non-covalent interaction between human cytomegalovirus protein IE1 and SUMO1 through two SIMs is critical for the transactivation activity of the viral protein. One of the best examples of functional interplay between SIMs and SUMOylated proteins is found in PML-nuclear bodies, dynamic structures that have a key role in immune response to invading pathogens. How SUMOylation can determine the outcome of the interplay between PML-NBs and pathogens has been reviewed by Patra and Müller.

SUMOylation can crosstalk with other post-translational modifications such as ubiquitination or ISGylation and act in concert to modulate protein function and signaling pathways. Chelbi-Alix and Thibault reviewed the implication of the crosstalk between SUMO and other ubiquitin-like proteins for antiviral defense.

SUMOylation plays also an essential role in the regulation of immune response in insects as shown by Nayak et al., who demonstrate that the SUMOylation of Arginyl tRNA synthetize modulates the Drosophila innate immune response. SUMOylation is also critical for plant response to environmental changes and during pathogen attack. The role of SUMOylation in plant immunity and the pathogenic strategies targeting plant SUMOylation have been reviewed by Sharma et al. SUMOylation level of different proteins can be regulated by only one protein. The report of Kasera et al. showed how losing the negative regulator of basal defenses rps4-RLD1 (SRFR1) increases global SUMOylation, suggesting that SRFR1 plays a critical role in maintaining SUMOylation homeostasis.

In conclusion, the contributions to this Research Topic highlight the fundamental role of SUMOylation in innate immunity of eukaryotic organisms.

## Author Contributions

CR: wrote this brief overview.

## Conflict of Interest

The author declares that the research was conducted in the absence of any commercial or financial relationships that could be construed as a potential conflict of interest.

## Publisher's Note

All claims expressed in this article are solely those of the authors and do not necessarily represent those of their affiliated organizations, or those of the publisher, the editors and the reviewers. Any product that may be evaluated in this article, or claim that may be made by its manufacturer, is not guaranteed or endorsed by the publisher.

